# Acetate Ringer’s solution versus 0.9% saline for septic patients: study protocol for a multi-center parallel controlled trial

**DOI:** 10.1186/s13063-020-05007-5

**Published:** 2021-01-25

**Authors:** Fang Liu, Jing Zhang, Yuan Zhu, Lianjiu Su, Yiming Li, Li He, Li Yu, Zhiyong Peng

**Affiliations:** 1 Department of Critical Care Medicine, Zhongnan Hospital of Wuhan University, 169 Donghu Road, Wuhan, 430071 Hubei China; 2 Department of Critical Care Medicine, Wuhan Central Hospital, Huazhong University of Science & Technology, Tongji Medical School, Wuhan, 430030 Hubei China

**Keywords:** Sepsis, Septic shock, Acute kidney injury, Saline, Acetate Ringer’s solution

## Abstract

**Background:**

Previous study drew different conclusions on significant differences between saline and balanced crystalloid solution infused in critical illness but both showed a statistical difference in the sepsis subgroup. Thus, we will specifically focus on septic patients in this study to compare the effects of saline and balanced solution. We hypothesize that effects of saline on renal outcomes are related to the underline acute kidney injury (AKI) severity and total volumes of infusion.

**Methods/design:**

The investigators designed a pragmatic, multi-center parallel controlled trial recruiting 312 patients who are diagnosed with sepsis/septic shock in the intensive care unit (ICU) and will be assigned with either acetate Ringer’s solution or saline in the corresponding month. Patients with an end-stage renal disease (ESRD) or who need renal replacement therapy (RRT) prior to or at the time of enrolment are excluded. Enrolled patients will be regarded as with mild, moderate, or severe sepsis on the basis of the severity of their illness and will be divided into subgroups according to their initial renal function and various intravenous infusion volumes when being analyzed. The primary outcome is major adverse kidney events within 28 days (MAKE28), including the composite of in-hospital death, receipt of new renal replacement therapy, or persistent renal dysfunction. Secondary outcomes include 28-day mortality, internal environment disturbance, incidence and duration of vasoactive drug treatment, duration of mechanical ventilation, duration of RRT, and ICU and hospital length of stay.

**Results and conclusions:**

To our knowledge, this study will be the first to focus on septic patients and provide credible and evident data on the comparison of outcome between acetate Ringer’s solution and saline for intravenous infusion in adult septic patients on the basis of baseline renal function and infusion volumes taken into consideration.

**Trial registration:**

ClinicalTrials.gov NCT03685214. Registered on August 15, 2018

**Supplementary Information:**

The online version contains supplementary material available at 10.1186/s13063-020-05007-5.

## Background and rationale

Sepsis, a common problem in the intensive care unit (ICU) with high morbidity and mortality [1–4], is the main cause for acute kidney injury (AKI) in critically ill adults [5]. Septic AKI accounts for nearly half of all kinds of AKI [6] and increases mortality six- to eightfold [6, 7]. Fluid resuscitation plays a vital role in the treatment of sepsis and septic shock [8, 9], which attaches great significance to the type of solution infused during fluid management. Saline is not a “normal” fluid with high level of chloride which may be related to AKI and mortality [10, 11], but it is still commonly administered on a global basis so far [12]. Meanwhile, a preference for balanced crystalloid solution on venous transfusion is emerging [13, 14].

Until now, the choice of crystalloid for critically ill patients has not been confirmed [15, 16]. The SPLIT (Saline v Plasma-Lyte 148 for Intensive Care Unit Fluid Therapy) study conducted in four New Zealand ICUs [15] concluded with no difference neither in the primary outcome of the incidence of AKI nor in secondary outcomes of RRT use and mortality between the use of a buffered crystalloid and saline. However, this study was criticized for the relatively small volume of fluid as it may have been too low to cause detectable renal toxicity [16]. Another trial comparing saline to buffered crystalloid solutions (lactated Ringer’s solution and Plasma-Lyte A) in a single ICU, the SALT (Isotonic Solution Administration Logistical Testing) study, demonstrated no difference in the overall incidence of AKI or major adverse kidney events (MAKE30) including death from any cause, new receipt of renal replacement therapy (RRT), or persistent renal dysfunction within 30 observational days. However, notably, there was a difference on MAKE30 in septic patients who received larger volume of crystalloids in subgroup analysis. The study showed a dose-response relationship [16]. A recent multiple-crossover trial in critically ill adults in 5 ICUs demonstrated that the use of balanced crystalloids resulted in a lower rate of MAKE30 than the use of saline [17] though it showed no significant difference in the incidence of AKI and mortality respectively. It also showed difference in the sepsis subgroup, but the potential dose-response relationship related with infusion volumes in the subgroup was not clarified [18]. Both of the 2 studies implied that it is necessary to investigate further in septic patients as they are more prone to fluid damage. It still remains uncertain whether balanced crystalloid is superior than saline in septic patients, whether it is related to their initial renal function, and whether there is a dose-response relationship between fluids and outcomes. Thus, we carry out this study for further investigations.

## Objectives

This trial is the first to focus on septic patients and verify if acetate Ringer’s solution is superior to saline, especially in patients with septic AKI. It is aimed to provide credible and evident data and clarify if (1) there is a significant difference in the overall outcome between acetate Ringer’s solution and saline in septic patients, (2) the effect is dependent on the baseline renal function, and (3) there is a dose-response effect by subgroup analysis.

## Methods/design

**Fig. 1:**
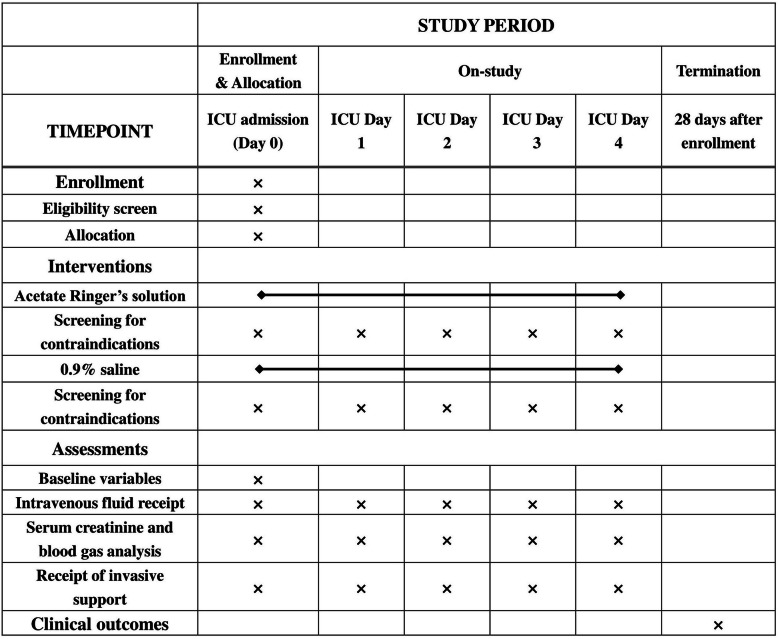
Standard Protocol Items: Recommendations for Interventional Trials (SPIRIT) checklist. Baseline variables include baseline renal function, main diagnosis and complications, severity of illness at enrolment, demographic characters, and admission location. Intravenous fluid includes saline, acetate Ringer’s solution, and other fluids. Receipt of invasive support includes mechanical ventilation, receipt of RRT, and vasopressors. Clinical outcomes include vital status, vasopressor days, mechanical ventilation days, RRT days, ICU stay days, hospital length of stay and serum creatinine at hospital discharge

**Fig. 2:**
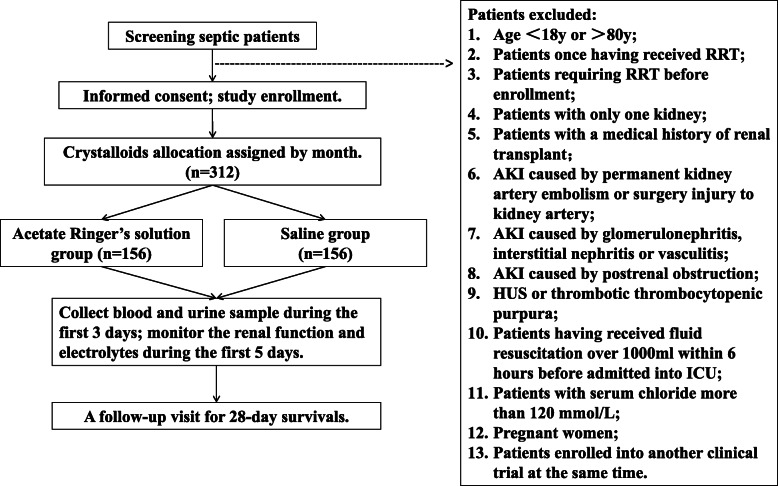
Study flow. Abbreviations: RRT, renal replacement therapy; AKI, acute kidney injury; HUS, hemolytic uremic syndrome; ICU, intensive care unit

The study is a multi-center, interventional, prospective, pragmatic [19], unblinded, and parallel controlled trial. This protocol is designed in accordance with the Standard Protocol Items: Recommendations for Interventional Trials (SPIRIT) guidelines [20]. All the data will be collected at each time spot as shown in the Standard Protocol Items (Fig. 1). The study flow is described in Fig. 2.

### Study sites and period

The study is planned to be conducted from March 1, 2019, to February 28, 2022, in 2 centers including the ICU of Zhongnan Hospital of Wuhan University and the ICU of Wuhan Central Hospital of Tongji Medical College, Huazhong University of Science and Technology. The above 2 hospitals are both tertiary hospitals integrating clinical, scientific research and teaching. Participating ICUs began enrolment sequentially over the first year of the study. Each ICU will enroll participants for an equal number of acetate Ringer’s solution and saline months for at least 12 months.

### Registration, ethical considerations, and monitoring

The study was registered on ClinicalTrials.gov before the participants’ enrolment started (identifiers: NCT03685214) (Table 1). This study follows the principles of the Helsinki Declaration 2013. The whole protocol has been reviewed and approved by the Ethics Committee of Zhongnan Hospital of Wuhan University (Clinical Ethical Approval No. 2018010) and the Ethics Committee of Wuhan Central Hospital of Tongji Medical College, Huazhong University of Science and Technology (Hospital Ethical Approval No. 201904). An independent data and safety monitoring board (DSMB) is monitoring the progress and safety of the trial. The DSMB is independent of the trial and is comprised of two academic intensivists, professor Jianguo Li and professor Bo Hu, who are experienced in the conduct of clinical trials in critical illness and outside the study, being able to pause the trial to investigate or give suggestions on potential safety issues to improve our design and implement. The DSMB follows the charter of the Ethics Committee of Zhongnan Hospital of Wuhan University.

**Table 1:** Trial registration information

Data category	Information
Primary registration and trial identifying number	ClinicalTrials.gov NCT03685214
Secondary identifying numbers	None
Source(s) of monetary or material support	Wu Jieping Medical Foundation (Project identifier: HRJJ20171026)
Primary sponsor	Zhongnan Hospital of Wuhan University
Secondary sponsor	Wu Jieping Medical Foundation
Contact for public queries	Zhiyong Peng, MD. PhD. [pengzy5@hotmail.com]
Contact for scientific queries	Zhiyong Peng, MD. PhD. [pengzy5@hotmail.com]
Public title	Comparison of Balanced Crystalloids and Normal Saline in Septic Patients
Scientific title	Acetate Ringer’s solution versus 0.9% saline for septic patients: study protocol for a multi-center parallel controlled trial
Countries of recruitment	China
Health condition(s) or problem(s) studied	Crystalloid infusion for septic patients
Intervention(s)	Acetate Ringer’s solution versus saline
Key inclusion and exclusion criteria	Inclusion criteria:
	1. At the age of 18 to 80
	2. Diagnosed as sepsis (a possible or specific proof for infection plus Sequential Organs Failure Assessment (SOFA) scores ≥ 2)
	Exclusion criteria:
	1. Patients once having received RRT
	2. Patients requiring RRT prior to enrolment
	3. Patients possessed with only one kidney
	4. Patients with a medical history of renal transplant
	5. AKI caused by permanent kidney artery embolism or surgery injury to kidney artery
	6. AKI caused by glomerulonephritis, interstitial nephritis, or vasculitis
	7. AKI caused by postrenal obstruction
	8. Hemolytic uremic syndrome (HUS) or thrombotic thrombocytopenic purpura
	9. Patients having received fluid resuscitation over 1000 ml within 6 h prior to ICU
	10. Patients with serum chloride more than 120 mmol/L
	11. Pregnant women
	12. Patients enrolled into another clinical trial at the same time
Study type	Parallel controlled trial
Date of first enrolment	March 2019
Target sample size	312
Recruitment status	Recruiting
Primary outcome(s)	MAKE28
Key secondary outcomes	1. The occurrence of AKI
	2. Twenty-eight -day mortality
	3. Electrolyte disturbance, including hypernatremia, hyperchloremia, and hyperkalemia as well as hyponatremia, hypochloremia, and hypokalemia
	4. Changes of renal functions based on the biomarkers measured from the participants’ plasma and urine samples collected in the first 3 days after enrolment
	5. Other clinical outcomes: ICU stay, ventilator days, vasopressor days, and RRT days

### Participant patients

Participants’ inclusion criteria are as follows:

1. At the age of 18 to 80;
2. Diagnosed as sepsis (a possible or specific proof for infection plus Sequential Organs Failure Assessment (SOFA) scores ≥ 2) [21].

Participants’ exclusion criteria are as follows:

1. Patients once having received RRT;
2. Patients requiring RRT prior to enrolment;
3. Patients possessed with only one kidney;
4. Patients with a medical history of renal transplant;
5. AKI caused by permanent kidney artery embolism or surgery injury to kidney artery;
6. AKI caused by glomerulonephritis, interstitial nephritis, or vasculitis;
7. AKI caused by postrenal obstruction;
8. Hemolytic uremic syndrome (HUS) or thrombotic thrombocytopenic purpura;
9. Patients having received fluid resuscitation over 1000 ml within 6 h prior to ICU;
10. Patients with serum chloride more than 120 mmol/l;
11. Pregnant women; and
12. Patients enrolled into another clinical trial at the same time.

### Study treatments

The intervention of treatment lies in fluid management. To exclude the possible difference caused by different balanced crystalloid solution, acetate Ringer’s solution is chosen for balanced crystalloid infusion. After patient screening and grouping, participants will be assigned with acetate Ringer’s solution or saline for intravenous infusion accordingly. The volume, infusion rate, and additive content (e.g., potassium chloride) of the fluid will be determined by responsible clinicians. The intervention will last for 5 days after patients’ enrolment. Other solutions are permitted to be used as carrier fluids for the infusion of any drug on the occasion when neither acetate Ringer’s solution nor saline is considered compatible. Medication use except fluids will not be restricted in the study.

### Parallel control and allocation

Patients will be divided into 2 parallel groups: acetate Ringer’s solution versus saline. Crystalloids for enrolled patients are assigned according to the number of the month. Once assigned, the solution will be applied for the first 5 days during the patients’ stay in ICU. The assignment remains during change of month. The solution applied in the beginning month in each ICU is determined randomly by Excel2016 of Microsoft Office to make sure that stratified patient groups will be randomized to receive either acetate Ringer’s or saline solution, followed by a monthly rotation of fluids, so that any potentially observed difference in renal function is not due to other confounding factors such as use of nephrotoxic medication or contrast media. According to the Excel2016-generated random numbers, acetate Ringer’s solution will be applied in the odd month and saline will be applied in the even month both in the 2 centers. The random number table is operated by the primary investigator alone and clinicians are not involved in the process.

### Study fluid distribution and logistics

Acetate Ringer's soution and saline administered in this trial both have different volume sizes of 250 ml and 500 ml. Components of these two crystalloids are presented (Table 2). The study is an open-label study; thus, the exact solution used is known to investigators, clinicians, and patients. Since the study is non-blind and two fluids used in this study have already been widely applied in the daily care of patients in the above ICUs, there are no problems of logistics.

**Table 2:** Components of two crystalloids administered in the trial

Components (mmol/L)	Acetate Ringer’s solution	0.9% saline
Na	140	154
K	4	-
Mg	1	-
Ca	1.5	-
Cl	115	154
Glucose (%)	1	-
Buffer system	Acetate 25	-
Osmotic concentration (mOsmol/kg)	304	286

### Study outcomes

The primary outcome is MAKE28 (major adverse kidney events) [22]—a composite of in-hospital death, new renal replacement therapy, or persistent renal dysfunction (defined by an estimate glomerular filtration rate (eGFR) lower than 60 ml/min/1.73 m^2^ for at least 3 months [23, 24]) within 28 observational days.

Secondary outcomes will include:

1. The occurance of AKI which is diagnosed according to the Kidney Disease Improving Global Outcomes (KDIGO) criteria [25];
2. Twenty-eight day mortality;
3. Electrolyte disturbance, including hypernatremia, hyperchloremia, and hyperkalemia as well as hyponatremia, hypochloremia, and hypokalemia;
4. Changes of renal functions based on the biomarkers measured from the participants’ plasma and urine samples collected in the first 3 days after enrolment; and
5. Other clinical outcomes: ICU stay days, ventilator days, vasopressor days, and RRT days.

Pre-specified subgroups for primary and secondary outcome analyses will mainly include:

1. With or without acute kidney injury (no AKI, AKI stage 1, AKI stage 2, or AKI stage 3);
2. With or without septic shock;
3. Low versus high severity of sepsis: mild, moderate, and severe (classified by SOFA scores or Acute Physiology and Chronic Health Evaluation II (APACHE II) scores); and
4. Intravenous infused volumes of the assigned crystalloids.

### Sample size and statistical power

The sample size was calculated based on the occurrence of MAKE30 on septic patients, which was around 35% [17]. With a noninferiority limit of 1.5%, a total of 312 study participants (156 in each group) would result in a power of at least 80% with a one-sided type-1 error rate (*α*) of 2.5%, allowing a 20% withdrawal rate in each group.

### Statistical analysis

Measurement data that conform to normal distribution will be described as mean ± SD, while ones that do not will be reported as the median and interquartile range (IQR). Count data will be notified as frequencies and proportions. As for single-factor analysis, the difference of measurement data will be compared with *T* test between two groups or with one-way analysis of variance among three groups. Chi-square test and Fisher’s exact test will be used for rate comparison. As for multi-factor analysis, variables which showed a significant difference in the univariate analysis will be processed by logistic multivariate regression analysis. Multiple linear regression analysis will be used to demonstrate the linear relationship between variables. *P* value < 0.05 will be considered statistically significant. SPSS 24.0 will be used to complete data processing and statistical analysis.

### Analytic rationale

This study will recruit participants with a wide range of baseline risk factors of the primary outcome who are exposed to the study intervention and can be divided into subgroups of distinct renal function or received crystalloid volumes. The baseline and secondary analysis will figure out whether the intervention makes a difference to patients’ prognosis.

### Primary analysis

The primary analysis will be an intention-to-treat comparison of the primary outcome of MAKE28 between the saline and acetate Ringer’s solution. A generalized linear mixed-effects model will be used including fixed effects (sex, age, body mass index, group assignment, principal diagnosis, crystalloids received prior to ICU, SOFA score, APACHE II score, mechanical ventilation status, vasopressor usage, etc.) and random effects. It is aimed to describe patients’ baseline characteristics and eliminate confounding factors.

### Main secondary analysis

We presume that participants will receive a wide range of total crystalloid volumes and that the more fluid patients receive, the more significant difference will be performed between the two groups. Also, we presume that the outcome may be related with participants’ initial renal function, which is defined according to KDIGO criteria [25] based on the fisrt creatinine or the urine output in the first 6, 12 or 24 hours after enrollment. Based on these anticipations, in the main secondary analysis, patients will be divided into several groups according to different crystalloid infusion volumes and distinct initial renal function, respectively. The proportion of patients experiencing the primary outcome will be compared between the same volume groups of saline and acetate Ringer’s solution respectively and between the same initial renal function groups, namely no AKI, AKI stage 1, AKI stage 2, and AKI stage 3, respectively. In this section, a logistic regression model with the primary outcome will be conducted to detect whether it differs significantly between saline and acetate Ringer’s solution infusion on renal outcome in the same volume group or the same initial renal function group, and then figure out whether the infused volume or initial renal function makes a difference.

### Additional secondary analysis

Statistical methods mentioned in the statistical analysis will be applied according to the characteristics and distribution of the data.

1. Compare the secondary outcome between the two groups;
2. Subgroup analysis will include:
  - Septic shock (yes/no)
  - Stage of AKI on the enrolment day (no AKI, AKI, chronic kidney disease without receiving RRT regularly)
  - intravenous volumes of the assigned crystalloids
  - The APACHE II scores and SOFA scores at the day of enrolment
  - Receipt of mechanical ventilation (yes/no) and ventilation days
  - Receipt of vasopressor (yes/no), vasopressor category, and max doses
  - Receipt of RRT (yes/no) and RRT days
  - Main diagnosis at the time of admission to hospital (nervou s system disease, respiratory system disease, cardiovascular system disease, digestive system disease, urinary system disease, hematologic system disease, endocrine system disease)
  - Complications (hypertension, diabetes, coronary heart disease, etc.)

### Handling of missing data

Of the primary outcome, data of the rate of AKI and the percentage of new receipt of RRT are not supposed to be missing for any patients. Nevertheless, some data of renal function may be missing due to protocol executive errors or record-keeping errors. Mean completer, hot deck imputation, and filling manually will be taken to minimize the effects of these missing data as much as possible. For example, if the renal function values near the missing value are in the normal range or have the same trend, the missing data will be averaged or filled with proximity. Patients without a serum creatinine measurement between enrolment and hospital discharge who survive and do not receive new RRT will be considered as not having experienced MAKE. If the patients or authorized agents give up treatment due to little chance to survive predicted by clinicians, their outcome is considered to be death. Besides, in-hospital mortality may be missing due to patients or authorized agents giving up treatment or other unpredictable accidents when the illness is not that severe. Data may also be missing due to severe adverse events (SAEs) or patients quitting the trial. Under these circumstances, an intention-to-treat analysis will be conducted to deal with the missing data. Of other outcomes, deletion will be considered if the missing data is too large to fill. Indices with complete data after conservative imputation will be included in the statistical analysis.

### Data collection and management

Patients’ demographic data, main diagnosis, comorbidities, assigned crystalloids, general vital signs, SOFA scores, APACHE II, infection indices, and indices of multi-organ function including renal function, respiratory function, cardiac function, etc. at the time of enrolment will be collected as baseline data. Clinical information including infection indices (white blood cell count, procalcitonin, etc.), organ function indices (renal function (creatinine, urea nitrogen, cystatin C, urine volumes, etc.), respiratory function (PO_2_/FiO_2_, ventilation situation, etc.), hepatic function (total bilirubin, etc.), and indices of internal environment (blood gas analysis) will be detected during the first 5 days in ICU, and treatments such as vasopressors, mechanical ventilation, RRT, and electrolyte supplement will be recorded. These data and infused volumes of the assigned fluids will be collected for at least 5 days or until ICU discharge. ICU stay days, vasopressor days, RRT days, and ventilation days will be counted during 28 days after enrolment. These data will be collected to assess and analyze primary and secondary outcomes. Besides, patients’ blood and urine samples within the first 3 days after enrolment will be collected and stored for testing possible biomarkers for identifying AKI at an early stage.

Patients’ information and clinical data will be collected from the Hospital Information System (HIS) of the hospitals involved in this study and recorded in the Case Report Format (CRF) by the trial manager or trained personnel. An electronic password-protected excel, which will be applied for statistical analysis, will be created to summarize the data of all participants. All the data will be accessed by only investigators and authorized personnel to monitor the completeness and authenticity of the table. The confidentiality is secured and all the data will be preserved for the purpose of a secondary analysis or investigations by the investigators. To protect patients’ privacy, their names will not appear on the CRF table. Every participant will only be recognized by their study ID.

### Risk evaluation and adverse events

The trial is considered to pose a low risk. Firstly, saline and acetate Ringer’s solution have already been widely used in the clinical practice of ICUs of the above hospitals. Secondly, it still remains controversial which fluid (saline or balanced crystalloid solution) is better for septic patients. Thirdly, clinicians are allowed to make clinical judgments and choose the other crystalloid for a specific patient if they think the assigned one may increase the risk of poor prognosis. Certainly, data of these patients will not be included in the final analysis. Therefore, the adverse events (AEs) of this trial may be minimal.

Still, during the entire observational 28 days from the beginning of the trial, possible AEs will be assessed and recorded in the CRF table. Investigators will evaluate the relationship between the events and our intervention by clinical judgment, and the events will be graded as mild, moderate, and severe. SAEs should be considered if unexpected clinically significant critically ill diseases which may be resulted from fluid intervention happen. AEs, especially SAEs, must be reported to DSMB and followed until they are solved. At the end of the trial, AEs and their relationship to the study will be documented in a table and submitted.

## Summary/discussion

Fluid resuscitation is the mainstream for the treatment of patients with sepsis/septic shock, and all the guidelines recommend crystalloids as the first choice. Normal saline, a very common crystalloid, poses hyperchloremia and probably worsens AKI which is always complicated by sepsis [26]. However, it is unknown if saline can be safely used in septic patients. Recent studies demonstrated different conclusions in kidney outcomes between saline and balanced solutions in overall critically ill patients [15–17], but showed a significant difference in septic patients by subgroup analysis [16, 17]. This enlightens us to study on a possible dose-response relationship in a high-risk population. Thus, we hypothesize that the harmful effects of saline are associated with the initial kidney function and infused volume of fluids. To confirm this hypothesis, we will carry out this study. As sepsis or septic shock is one of the most typical problems requiring large volume of fluids, this also makes it clinically practically significant to investigate. To our knowledge, this study will be the first to focus on septic patients and compare the effects on outcome between balanced crystalloid solution and saline. In light of this study, a particular comparison will be conducted between acetate Ringer’s solution and saline. Firstly, the patients recruited will be confined to septic/septic shock. Therefore, the conclusions will be applied specifically in septic population. Secondly, the analysis of the association between the due solution and outcome, especially the development of AKI, will be more in details. The infused volume and the baseline renal function will both be taken into consideration. If this trial demonstrates that balanced crystalloid solution or saline shows a priority over the other, it can provide an evidence for the guideline on fluid resuscitation for septic/septic shock. Therefore, the results of this study may be instructive and meaningful.

### Trial status

This is the second version of the protocol which was reviewed and approved on April 20, 2018. Patient recruitment has started on March 1, 2019, and will be completed on February 28, 2022.

## Supplementary Information

**Additional file 1.**

## Data Availability

The CRFs and final dataset will only be accessible to the study investigators.

## Abbreviations

AKI: Acute kidney injury
ICU: Intensive care unit
ESRD: End-stage renal disease
RRT: Renal replacement therapy
SPLIT: Saline v Plasma-Lyte 148 for Intensive Care Unit Fluid Therapy
SALT: Isotonic Solution Administration Logistical Testing
MAKE: Major adverse kidney events
SPIRIT: Standard Protocol Items: Recommendations for Interventional Trials
KDIGO: Kidney Disease Improving Global Outcomes
DSMB: Data and safety monitoring board
HUS: Hemolytic uremic syndrome
SOFA: Sequential Organs Failure Assessment
eGFR: Estimate glomerular filtration rate
APACHE II: Acute Physiology and Chronic Health Evaluation II
IQR: Interquartile range
CRF: Case Report Format
AE: Adverse event
SAE: Severe adverse event
