# Supplementary material for: Acetate Ringer’s solution versus 0.9% saline for septic patients: study protocol for a multi-center parallel controlled trial
Source: Trials. 2021 Jan 25;22:89. doi: 10.1186/s13063-020-05007-5 (PMC7830046; doi:10.1186/s13063-020-05007-5)
Supplement: Supplementary file 1 — Additional file 1. [file 13063_2020_5007_MOESM1_ESM.doc]

**知情同意书**

| 项目名称 | 醋酸林格式液与生理盐水对ICU脓毒症患者的预后比较 |
| --- | --- |
| 研究单位 | 武汉大学中南医院 |
| 主要研究者 | 彭志勇 |
| 知情同意书版本号 | V2.0 |
| 知情同意书版本日期 | 2018年4月20日 |

您将被邀请参加一项临床研究。本知情同意书提供给您一些信息以帮助您决定是否参加此项临床研究。请您仔细阅读，如有任何疑问请向负责该项研究的研究者提出。

您参加本项研究是自愿的。本次研究已通过本院医学伦理委员会审查。

研究目的：目前关于平衡盐溶液与生理盐水在脓毒症的应用利弊存在争议。本研究通过探究平衡盐溶液相比于生理盐水在ICU应用，明确两者对脓毒症患者肾功能及其预后的影响，为当前脓毒症患者的液体管理策略提供临床实验支持。

研究过程：研究中，我们将予以对比使用平衡盐溶液患者与使用生理盐水患者在ICU的肾功能变化及预后差异。患者将会随机为两组：使用平衡盐溶液注射组及使用生理盐水注射组。该两组患者除复苏液体使用不同外，无其他任何监护及治疗差异。期间我们会从患者治疗需要复查的血液、尿液等检验样本中抽取少量样本进行该临床研究。患者出院后我们将会有1次电话随访。如果您同意参与这项研究，我们将和您或您的家人进行详细沟通，向您介绍该项研究的有关情况，也请您提供与疾病有关的情况，包括发病过程、家族史、以前就诊情况及曾经做过一些检查结果等。我们将对每位参与者进行编号，建立病历档案。在研究过程中我们需要采集一些您的标本，将由专业人员为您取样，例如从您已建立的血管通路中抽取动、静脉血2-3毫升，并留取尿液10毫升，共需10次。该操作不会增加受试者的任何痛苦和不适。您的样品仅用于本项研究。

风险与不适：对于您来说，与我们进行沟通、交谈可能会有些心理不适。所有的信息将是保密的。您的样本采集将严格按照无菌要求操作，标本的采集可能会有一些非常小的风险，包括短暂的疼痛、局部青紫，少数人会有轻度头晕，或极为罕见的针头感染。

受益：通过对您的信息资料进行研究，将为您的治疗提供必要的建议，或为疾病的研究提供有益的信息。

作为研究受试者，您有以下职责：提供有关自身病史和当前身体状况的真实情况；告诉研究医生自己在本次研究期间所出现的任何不适；告诉研究医生自己在最近是否曾参与其他研究，或目前正参与其他研究。

隐私问题：如果您决定参加本项研究，您参加试验及在试验中的个人资料均属保密。负责研究医师及其他研究人员将使用您的医疗信息进行研究。这些信息可能包括您的姓名、地址、电话号码、病史及在您研究来访时得到的信息。您的档案将保存在有锁的档案柜中，仅供研究人员查阅。为确保研究按照规定进行，必要时，政府管理部门或伦理审查委员会的成员按规定可以在研究单位查阅您的个人资料。这项研究结果发表时，将不会披露您个人的任何资料。

您可以选择不参加本项研究，或者在任何时候通知研究者要求退出研究，您的数据将不纳入研究结果，您的任何医疗待遇与权益不会因此而受到影响。

如果您需要其它治疗，或者您没有遵守研究计划，或者发生了与研究相关的损伤或者有任何其它原因，研究医师可以终止您继续参与本项研究。

您可随时了解与本研究有关的信息资料和研究进展，如果您有与本研究有关的问题，或您在研究过程中发生了任何不适与损伤，或有关于本项研究参加者权益方面的问题您可以通过联系电话027-67811773与重症医学科彭志勇主任联系。

如果您对您的权益有任何问题，您可以在国家法定工作日的工作时间联系:

武汉大学中南医院医学伦理委员会

联系电话：027-67812787

**知情同意书**

我已经阅读了本知情同意书。

我有机会提问而且所有问题均已得到解答。

我理解参加本项研究是自愿的。

我可以选择不参加本项研究，或者在任何时候通知研究者后退出而不会遭到歧视或报复，我的任何医疗待遇与权益不会因此而受到影响。

如果我需要其它治疗，或者我没有遵守研究计划，或者发生了与研究相关的损伤或者有任何其它原因，研究医师可以终止我继续参与本项研究。

我将收到一份签过字的“知情同意书”副本。

受试者签名：_________________________

受试者监护人签名：____________________

联系电话（手机）：_________________

日期：_____年____月_____日

我已准确地将这份文件告知受试者，他/她准确地阅读了这份知情同意书，并证明该受试者有机会提出问题。我证明他/她是自愿同意的。

研究者姓名：________________________

研究者签名：_________________________

联系电话（手机）：____________________

日期：________年________月________日

（注：如果受试者不识字时尚需见证人签名，如果受试者无行为能力时则需监护人签名）

English translation:

Informed Consent

| Project Name | Acetate Ringer’s solution versus 0.9% saline for septic patients |
| --- | --- |
| Research Unit | Zhongnan Hospital of Wuhan University |
| Primary Investigator | Zhiyong Peng |
| Version | V2.0 |
| Date | April 20, 2018 |

You will be invited to participate in a clinical study. This informed consent form provides you with some information to help you decide whether to participate in this clinical study. Please read it carefully. If you have any questions, please ask the researcher in charge of the study.

Your participation in this study is voluntary. This study has been reviewed by the Medical Ethics Committee of our hospital.

Research purpose: It remains controversial whether there is a difference for applying balanced crystalloid solution or normal saline in septic patients. This study aims to investigate the difference between balanced crystalloid solution and saline, clarify the impact of the two on the prognosis, especially on the renal outcome, of patients with sepsis, and provide clinical reference for the current fluid management strategy for septic patients.

Research process: Patients will be divided into two groups: a balanced crystalloid solution group and a saline group. Monitoring and treatment except for fluid management will not be intervened. During the research, some blood and urine specimens will be collected from your clinical samples for laboratory tests. You will receive a follow-up call after discharging. If you agree to participate in this research, we will introduced this trial and communicate in detail with you or your family members. You are supposed to provide information related to your disease, including the onset, past medical history, family history, previous testing findings and treatments, etc. We will number each participant and create an individual medical record file. During the research process, sample collection is necessary and will be operated by professionals. For example, take 2-3 ml of arterial or venous blood from your established vascular access, and collect 10 ml of urine. Ten times in total. This operation will not add any pain and discomfort to the subject. Your sample is only used for this study.

Risks and discomfort: For you, communicating with us may cause some psychological discomfort. All of your private information will be kept confidential. Your sample collection will strictly follow the sterile requirements. The sample collection may cause some low risks, including short-term pain, temporary bruising, few people will have mild dizziness, or rare needle infections.

Benefits: Research on your clinical documents may provide you with necessary advice for treatment or provide useful information for disease research.

As a research subject, you have the following responsibilities: provide the actual information about your medical history and current physical condition, tell researchers about any discomforts you have experienced during this study, and whether you have participated in other studies recently or currently.

Privacy issues: If you decide to participate in this study, your personal information about in the trial will be kept confidential. Your information will only be used for this research. Collected information may include your name, address, telephone number, medical history, and clinical information obtained during your research visit. Your files will be kept in a locked filing cabinet, which is only available to researchers. To ensure that the research is conducted in accordance with the regulations, when necessary, relevant government departments or the Ethics Committee can consult your personal data in the research unit according to the regulations. When the results of this research are published, no personal information about you will be disclosed.

You can choose not to participate in this research, or notify the researcher to withdraw from the research at any time. Your data will not be included in the research results, and any of your medical treatment and benefits will not be affected.

If you need other treatments, do not follow the study plan, or have a study-related injury, or if there is any other reason that the researcher think will affect the quality of the trial, researchers can terminate your continued participation in this study.

You can keep abreast of the information and progress related to this research. If you have any questions about this study, any discomfort or injury during the research, or any questions about the rights of participants, you can contact Peng Zhiyong, director of the Department of Critical Care Medicine, at 027-67811773.

If you have any questions about your rights, you can contact us during working hours on national legal working days:

Medical Ethics Committee of Zhongnan Hospital of Wuhan University

Contact number: 027-67812787

Informed consent

I have read this informed consent form.

I have the opportunity to ask questions and all questions have been answered.

I understand that participation in this study is voluntary.

I can choose not to participate in this research, or I will withdraw after informing the researcher at any time without being discriminated against or retaliated. Any of my medical treatment and benefits will not be affected due to my withdrawal.

If I need other treatment, or if I do not follow the research plan, or there is a research-related injury, the research physician can terminate my continued participation in this research.

I will receive a signed copy of the "Informed Consent".

Subject's signature: _________________________

Signature of Subject’s Guardian: ____________________

Contact number (mobile phone): _________________

Date: ______________

I have accurately informed the subject of this document, he/she accurately read the informed consent form, and proved that the subject has the opportunity to ask questions. I certify that he/she consented voluntarily.

Investigator’s name: ________________________

Investigator’s signature: _________________________

Contact number (mobile phone): ____________________

Date: ________________

(Note: If the subject is illiterate, the witness’s signature is required, if the subject is incapacitated, the guardian’s signature is required)
